# Molecular Mechanisms in Genetic Aortopathy–Signaling Pathways and Potential Interventions

**DOI:** 10.3390/ijms24021795

**Published:** 2023-01-16

**Authors:** Charlotte Xue Dong, Cassandra Malecki, Elizabeth Robertson, Brett Hambly, Richmond Jeremy

**Affiliations:** 1 Faculty of Health and Medical Sciences, University of Sydney, Sydney, NSW 2006, Australia; 2 The Baird Institute, Camperdown, NSW 2042, Australia

**Keywords:** aorta, aneurysm, Marfan, endothelium, vascular smooth muscle

## Abstract

Thoracic aortic disease affects people of all ages and the majority of those aged <60 years have an underlying genetic cause. There is presently no effective medical therapy for thoracic aneurysm and surgery remains the principal intervention. Unlike abdominal aortic aneurysm, for which the inflammatory/atherosclerotic pathogenesis is well established, the mechanism of thoracic aneurysm is less understood. This paper examines the key cell signaling systems responsible for the growth and development of the aorta, homeostasis of endothelial and vascular smooth muscle cells and interactions between pathways. The evidence supporting a role for individual signaling pathways in pathogenesis of thoracic aortic aneurysm is examined and potential novel therapeutic approaches are reviewed. Several key signaling pathways, notably TGF-β, WNT, NOTCH, PI3K/AKT and ANGII contribute to growth, proliferation, cell phenotype and survival for both vascular smooth muscle and endothelial cells. There is crosstalk between pathways, and between vascular smooth muscle and endothelial cells, with both synergistic and antagonistic interactions. A common feature of the activation of each is response to injury or abnormal cell stress. Considerable experimental evidence supports a contribution of each of these pathways to aneurysm formation. Although human information is less, there is sufficient data to implicate each pathway in the pathogenesis of human thoracic aneurysm. As some pathways i.e., WNT and NOTCH, play key roles in tissue growth and organogenesis in early life, it is possible that dysregulation of these pathways results in an abnormal aortic architecture even in infancy, thereby setting the stage for aneurysm development in later life. Given the fine tuning of these signaling systems, functional polymorphisms in key signaling elements may set up a future risk of thoracic aneurysm. Multiple novel therapeutic agents have been developed, targeting cell signaling pathways, predominantly in cancer medicine. Future investigations addressing cell specific targeting, reduced toxicity and also less intense treatment effects may hold promise for effective new medical treatments of thoracic aortic aneurysm.

## 1. Background

Thoracic aortic aneurysm (TAA) is characterized by progressive aortic dilation, often asymptomatic until dissection or rupture occurs. One in five TAA result from gene mutations affecting vascular smooth muscle cells (VSMC) or the extracellular matrix (ECM) [1]. Genetic conditions predisposing to TAA include Marfan syndrome (MFS), Loeys-Dietz syndrome (LDS) and heritable non-syndromal aneurysm (hTAA) [2,3]. In addition, TAA is also commonly associated with bicuspid aortic valve (BAV) [4].

Pathological features of TAA include ECM degradation, VSMC dedifferentiation, inflammation and endothelial cell (EC) dysregulation [5,6,7]. These features are driven by changes in cell signaling, with the dysregulation of key structural and functional elements in the aorta [8]. Signaling pathways involve cascades of protein activation and inhibition, which control cell function and destination, proliferation, migration and apoptosis. Crosstalk exists between EC and VSMC and between pathways, supporting complex functional interactions, which may be antagonistic or synergistic [9,10].

The transforming growth factor β (TGF-β) signaling pathway has been the focus of investigation for genetic TAA [11,12]. Both TGF-β and mitogen-activated protein kinase/extracellular signal-regulated kinase (MAPK/ERK) signaling can act in synergy to promote ECM degradation and remodeling [2,13]. Other pathways, including Angiotensin, WNT, NOTCH and PI3K/AKT signaling, may also contribute to TAA [14,15]. Individual signaling pathways may act as up- or down-stream effectors and/or work in parallel with other pathways to co-regulate VSMC and ECM homeostasis. Dysregulation in one pathway may result in changes of activity of multiple other pathways, with the accumulation of aberrant signaling ultimately leading to TAA.

Deciphering the contributions of intersecting signaling pathways to TAA is key to developing effective therapeutics. As a fundamental step towards supporting future studies, this review examines the evidence for the roles of different cell signaling pathways in TAA. The functions of each pathway in VSMC and EC, and interactions between pathways, are described. Evidence for the involvement of each pathway in the pathogenesis of TAA is reviewed and finally, potential therapeutic interventions are examined.

## 2. Cell Function in Normal and Aneurysmal Aorta

The principal anatomical layers of the aorta are the intima, media and adventitia. The intima is a monocellular layer of ECs, separating the bloodstream from the inner aortic wall, and includes a basement membrane, subendothelium and internal elastic membrane. The intima has an essential role in vascular homeostasis, through ion and solute exchange and hemodynamic signaling. The media lies between the intima and outer adventitia, with elastin laminae, type I & III collagen, fibrillin-1 microfibrils and proteoglycans, forming the ECM that surrounds the VSMC, to form the lamellar units [2]. The media modulates response to pulsatile hemodynamic load [16]. The VSMCs are normally in a quiescent, contractile phenotype, with low levels of synthetic activity or proliferation. The outer adventitia mainly consists of type I & III collagen fibers, the vasa vasorum, which penetrate to the media, and perivascular nerves between loosely arranged fibroblasts. The adventitia supports static load-bearing [17].

Pathological features of TAA include the degradation of the ECM, the loss of VSMC due to apoptosis, and the fragmentation of elastin laminae [5,6,7,18,19,20]. The VSMC in TAA are characterized by a phenotypic switch from contractile (differentiated) to synthetic (dedifferentiated) form, with cellular proliferation, and the expression of proteolytic enzymes including matrix metalloproteinases (MMP) [21,22,23]. Contractile VSMC markers, including smooth muscle actin (SMA), smooth muscle protein 22-alpha (SM22α), calponin, and vimentin, are decreased in TAA. Synthetic VSMC promote inflammation, elastin degradation, vascular fibrosis, and calcification, leading to arterial stiffening [24].

Normal endothelium promotes vasodilation via nitric oxide (NO), contributes to the inhibition of VSMC proliferation and migration, and also inhibits platelet aggregation and adhesion [25]. In contrast, when challenged, EC become activated, pro-thrombotic and adhesive, and can also influence VSMC phenotype and exacerbate TAA, although mechanisms remain to be fully explained [26]. Low NO availability may impair signaling between EC and VSMC and is associated with an inflammatory response to adverse stimuli [27,28,29,30]. There are conflicting data regarding EC proliferation and migration in TAA. In genetic TAA, EC show increased migration and proliferation with neo-angiogenesis and ECM remodeling, especially in MFS [18]. In contrast, other studies have observed a lower proliferation of EC in TAA associated with BAV, compared to controls or TAA associated with normal tricuspid aortic valves (TAV) [26].

## 3. TGF-β Signaling

The most recognized pathway associated with TAA is the TGF-β cascade [31,32,33]. The TGF-β signaling family is a highly conserved group of ligands and cell receptors, contributing to cell growth, differentiation, homeostasis and apoptosis. Signaling by TGF-β involves coupled intracellular pathways, specifically SMAD-dependent (canonical) pathways and SMAD-independent (non-canonical) pathways, including PI3K/AKT and MAPKs [34,35]. Conditions associated with disordered TGF-β signaling include MFS, LDS and also systemic sclerosis, diabetic glomerulosclerosis and some cancers [36].

There are multiple ligands in the TGF-β family, including TGF-β and bone morphogenic proteins (BMPs). Receptors are heterodimers of Type II (TGF-β and BMP subtypes) and Type I receptors, with the latter having several subtypes, notably Activin-like kinase 5 (ALK5) and Activin-like kinases 1/2/3/6 (ALK1/2/3/6). The ALK5 receptors transduce TGF-β signaling to activate SMAD2/3, whilst ALK1/2/3/6 transduce BMP signaling to activate SMAD1/5/8 [37]. Signaling by TGF-β/BMP results in the transcriptional activation of multiple target genes, the actual mix of which is determined by the specific intracellular pathway activated by ligand-receptor binding. Key transcription factors involved in SMAD signaling include the AP-1 factors, RUNX, FOX and SP1, whilst non-canonical signaling via the MAPKs JNK, p38 or ERK involves c-Jun, c-Fos or c-Myc, respectively. 

### 3.1. Regulation of TGF-β Signaling

There are several layers of regulation of TGF-β signaling [38]. The first is the availability of TGF-β ligand, which is tightly regulated by sequestration of free TGF-β to a latent binding protein (LTBP), itself associated with structural elements of the intracellular matrix, including fibrillin microfibrils. In the extracellular space, the interaction of growth factors, e.g., connective tissue growth factor (CTGF), with TGF-β is associated with increased TGF-β/ALK5 binding, but interaction of CTGF with BMP reduces BMP/ALK1 binding [39].

The second level of regulation involves internalization and the recycling or destruction of activated receptors. Interestingly, some receptors are specifically associated with cholesterol-rich regions of the cell membrane and these receptors are particularly associated with activation of MAPK/ERK by TGF-β. Furthermore, the depletion of cholesterol in these regions appears to inhibit TGF-β induction of MAPK/ERK signaling [40]. The degradation of receptors is via ubiquitination, involving SMURF1, SMURF2 and NEDD-4, with SMAD7 binding as a key cofactor. As an expression of SMAD7 is itself induced by TGF-β/BMP, there is a potential functional feedback loop through receptor endocytosis and degradation.

Subsequent to the receptor level, the availability and activation of SMADs are also regulated. The binding of regulatory SMADs to the receptors is antagonized by SARA and BAMBI [41], whilst SMAD4 is the key intracellular regulating partner for active signaling. In addition, SMAD7 can inhibit the DNA binding of the SMAD2/3/4 complex. Feedback mechanisms for the regulation of TGF-β signaling also exist. The SMAD co-repressor SNON is encoded by the SKI-like gene *SKIL*, the expression of which is increased by TGF-β [42].

### 3.2. TGF-β and VSMC

**Figure 1:**
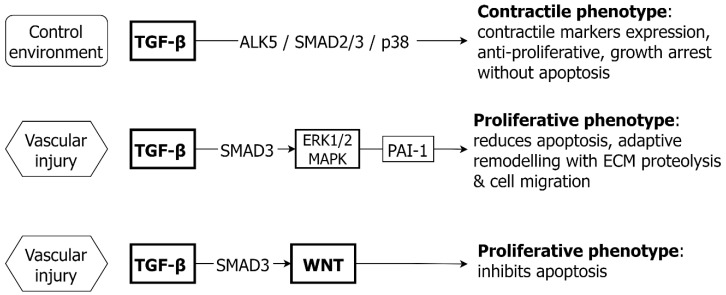
Summary of the mechanisms of TGF-β signaling pathways in VSMC. In the control environment, TGF-β supports the contractile phenotype by signaling through ALK5/SMAD2/3/p38. Following vascular challenge, TGF-β stimulation in presence of increased SMAD3 is associated with transactivation of EGFR and activation of ERK1/2/MAPK/PAI-1, resulting in change is phenotype with secretion of MMP and ECM remodeling. Another injury response in presence of increased SMAD3 is receptor independent activation of WNT/β-catenin, with associated reduction in VSMC apoptosis.

TGF-β can have bidirectional effects on VSMC, either promoting the contractile phenotype and maintaining normal vascular architecture [43,44] or promoting the proliferation and migration of the VSMC, with vascular remodeling in response to injury [45,46,47,48] (Figure 1).

In the developing vasculature, TGF-β signaling is required for the normal differentiation of VSMC from stem cells [49]. The loss of TGF-β signaling is associated with abnormal vascular development and disordered functional responses in the aorta [50]. Similarly, in the adult aorta TGF-β signaling has a key role in maintaining the normal contractile phenotype of VSMC. In isolated murine aortic VSMC, TGF-β stimulation promotes growth arrest without apoptosis, leading to an overall anti-proliferative response. The signaling pathway includes the TGF-β receptor (TGFBR1/2), SMAD2/3 and p38 [51]. In both rodent and human VSMCs, TGF-β enhances the expression of contractile markers including SM-22α and SM-major histocompatibility complex [52].

The anti-proliferative effects of TGF-β in VSMC can be overcome in vascular injury, accompanied by excess SMAD3, or the TGFBR1 associated activation of EGFR, with effect via the ERK/MAPK pathway [45,53,54]. Both ERK/MAPK and SMAD3 activation can result in an increased expression of plasminogen activator inhibitor type-1 (PAI-1), which is associated with both increased VSMC proliferation and reduced apoptosis [55]. The binding of PAI-1 to cell surface receptors (e.g., low density lipoprotein receptor-related protein-1) regulates ECM proteolysis and remodeling, in addition to cell migration [56]. In turn, blockade of ERK/MAPK, can prevent this paradoxical proliferative response to TGF-β [45]. Another potential mechanism of VSMC proliferation in the presence of excess SMAD3 is via crosstalk with WNT/β-catenin signaling, with the upregulation of WNT proteins and stabilization of β-catenin, the latter supporting a proliferative response [57].

### 3.3. TGF-β and EC

**Figure 2:**
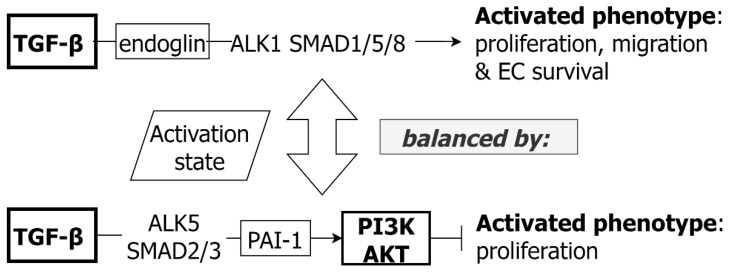
Summary of the mechanisms of TGF-β signaling pathways associated with activation and proliferation of EC. TGF-β activates EC by an endoglin/ALK1/SMAD1/5/8 pathway while EC activation is inhibited by the ALK5/SMAD2/3/PAI-1/PI3K/AKT pathways.

As with VSMC, TGF-β signaling in EC is required for the maintenance of vascular integrity and TGF-β can have bidirectional effects on ECs according to specific receptor/pathway activation (Figure 2). Two TGF-β type-1 receptors, Activin-like kinases 1 and 5 (ALK1 or ACVRL1, ALK5 or TGFBR1), are involved in TGF-β EC signaling and may play opposite roles, with ALK1 (expressed in EC) signaling through SMAD1/5/8, and ALK5 (expressed in EC and VSMC) signaling via SMAD2/3. The ALK1/SMAD1/5/8 axis induces EC proliferation and migration, and also promotes EC survival, via increased downstream Id1 (Inhibitor of DNA binding 1) expression [58]. In contrast, the ALK5/SMAD2/3 axis results in increased PAI-1 levels, probably partly via PI3K-AKT co-signaling [30,59,60,61]. The balance of ALK1vs ALK5 signaling contributes to determination of EC state (quiescent or active) [58,59]. Of note, the TGF-β co-receptor, endoglin, is required for the maintenance of ALK1 signaling and the absence of endoglin results in the suppression of EC proliferation [62].

## 4. WNT Signaling

The WNT pathway is complex and evolutionarily conserved, with a fundamental role in organ development [63] and also adult cell homoeostasis, influencing cell survival, proliferation and migration [64]. Detailed reviews of the WNT pathway are available [65,66,67]. Of the 19 known human WNT signaling ligand genes, those expressed in the human heart and vasculature include *WNT2B, WNT4, WNT5A, WNT5B, WNT10B* and *WNT11* [68]. In addition to these multiple ligands, there are multiple Frizzled (FZD) receptors and co-receptors, with Frizzled-1 (for WNT1, WNT2, WNT3, WNT3a, WNT8), Frizzled-2 (for WNT5a) and Frizzled-5 (for WNT2, WNT5a, WNT7a, WNT8, WNT11) known to be expressed in VSMC or EC, whilst the best characterized co-receptor is low-density lipoprotein receptor-related protein 5/6 (LRP5/6).

Different WNT ligands may activate canonical or non-canonical signaling with opposing results, whilst one pathway may also exert negative regulatory effects upon the other [69]. The activation of canonical WNT signaling is associated with increased cytoplasmic β-catenin, with resulting co-activation of TCF/LEF transcription factors in the nucleus. The activation of β-catenin and TCF/LEF is associated with the altered expression of cell-cycle-related proteins in VSMC, i.e., cyclin D1 and p21 [70]. Non-canonical WNT signaling is β-catenin independent and involves a Ca^++^ dependent pathway or small GTPases, which activate transcription factor nuclear factor of activated T cells (NFAT) and JUN-N terminal kinase (JKN), respectively. The main role of non-canonical signaling is regulating cellular polarity and migration [71].

### 4.1. Regulation of WNT Signaling

The WNT pathway is regulated at multiple levels, beginning with receptor-ligand binding. The effects of WNT are modulated by changes in Frizzled receptor type and expression on the cell surface [72]. The key intracellular regulator of WNT/β-catenin signaling is the Axin/GSK3-β destruction complex, which mediates phosphorylation and ubiquitination of β-catenin, leading to its degradation and the maintenance of the WNT/β-catenin in a quiescent or “OFF” state. Multiple proteins can influence the activity of WNT signaling, with inhibitors including WIF-1, NOTUM and TRABD2B, which target WNT [73,74]. Other inhibitors include DKKs and SOST, which target LRP5/6 [75], and IGFBP-4 and ZNRF3/RNF43 ubiquitin ligases, which target FZD for degradation [76,77]. In turn, ZNRF3/RNF43 is inhibited by R-Spondin, resulting in the activation of WNT signaling. Other activators of WNT signaling include Syndecan-1, acting through R-Spondin [78,79].

### 4.2. WNT and VSMC

**Figure 3:**
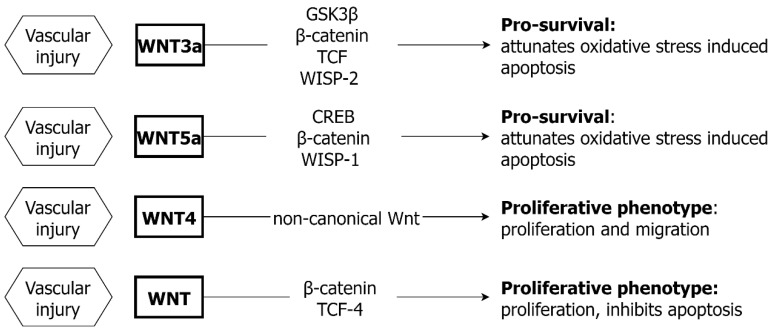
Summary of the mechanisms of WNT signaling pathways in VSMC. Overall, WNT signaling promotes VSMC survival, with non-canonical and β-catenin/TCF4 signaling also promoting VSMC proliferation.

The activation of WNT signaling promotes VSMC survival in the adult aorta (Figure 3). Both direct and indirect effects upon cell survival are consequent upon β-catenin and downstream transcription factors [64,80]. In experimental studies, the inhibition of β-catenin in arterial VSMC increases cell apoptosis, and conversely WNT5a stimulation attenuates oxidative-stress induced VSMC apoptosis [64,81]. Under hypoxic conditions, both WNT5a and WNT3a attenuate VSMC apoptosis induced by H_2_O_2_ [82]. The anti-apoptotic effects of WNT5a diminish with age due to the loss of CREB activation, but WNT3a effects are not impaired, reflecting different downstream targets. Specifically, WNT3a promotes VSMC survival via a GSK3β/β-catenin/TCF/WISP-2 dependent pathway, whilst WNT5a acts via CREB/β-catenin/WISP-1 signaling [83]. Furthermore, in human vasculature, WNT can promote VSMC proliferation and migration [84], through non-canonical signaling [64,85,86,87] (Figure 3). Additionally, WNT4, a non-canonical ligand, has been shown to be a key ligand to stimulate VSMC proliferation and intimal thickening in atherosclerosis [64,80,87]. Other studies have reported that WNT/β-catenin may induce venous SMC proliferation and migration by IL-8 stimulation through WISP-1 [88]. As with TGF-β, WNT signaling influences VSMC survival after injury [81]. The expression of β-catenin is elevated in a TCF-4-dependent manner after balloon injury, inhibiting VSMC apoptosis and stimulating proliferation [89,90].

### 4.3. WNT and EC

**Figure 4:**
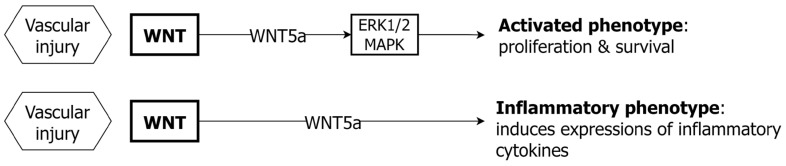
Summary of the mechanisms of WNT signaling pathways in EC. Under vascular injury, WNT5a and ERK1/2/MAPK dependent signaling leads to an activated phenotype and can promote an inflammatory phenotype.

The WNT pathway also mediates multiple signaling outcomes in EC, including EC proliferation and migration, inflammation and EC responses to external stimuli (Figure 4). Beyond its role in embryonic development, WNT continues to influence angiogenesis and EC function in later life [91]. In isolated human primary EC, WNT5a induces non-canonical signaling, with the induction of ERK phosphorylation, and the suppression of canonical WNT signaling [92]. The exposure of isolated human EC to WNT5a is associated with increased EC proliferation and survival, whilst the blockade of WNT5a in HUVECs is associated with reduced cell proliferation [93]. In cultured HAECs, WNT5a induces expression of COX-2 and other inflammatory cytokines including IL-8, IL-6 and NF-kB, with the mechanism of WNT5a signaling being via a Ca^++^ dependent (non-canonical) mechanism [94]. It appears that WNT5a, along with tumor necrosis factor (TNF) can modulate an inflammatory EC phenotype. These differing experimental findings indicate that EC responses to WNT signaling are cell-type and context dependent.

## 5. PI3K/AKT Signaling

The intracellular PI3K/AKT/mTOR pathway influences multiple cellular processes, including the cell cycle, protein synthesis, glucose metabolism and inflammation. As a result, PI3K/AKT plays key roles in cell survival, proliferation, growth, differentiation and migration. Among the downstream effects of PI3K/AKT is phosphorylation and inhibition of FOXO, itself a stimulus to apoptosis [95].

Several activators of PI3K/AKT are known, including growth factors binding to receptor tyrosine kinases (RTKs), TNF-α, oxidised LDL cholesterol and glycation end-products. Ligand-RTK binding activates PI3K, which catalyses phosphorylation of PIP_2_ to PIP_3_, which binds to and activates AKT. There are three isoforms (AKT1, AKT2, AKT3), with differing tissue expression and roles. Thus, AKT1 particularly influences cell survival and proliferation, while AKT2 influences VSMC contractile phenotype [96,97]. Phosphorylated AKT interacts with multiple intracellular intermediates, including CREB, p27, FOXO and mTOR. The PI3K/AKT pathway is involved in many diseases, including cancer, cardiovascular disease (CVD) and immune disorders. The activation of PI3K/AKT in VSMC and EC contributes to angiogenesis and vascular homeostasis [98,99]. A detailed review of PI3K/AKT signaling is available [100].

### 5.1. Regulation of PI3K/AKT Signaling

Given its role as an intracellular effector pathway, the primary regulators of PI3K/AKT activity are binding of growth factors to RTK receptors, which processes are subject to ligand concentration and receptor density. The activity of PI3K is antagonized by the tumor suppressor protein phosphatase and tensin homolog (PTEN), which dephosphorylates PIP_3_. Other regulators of AKT activation include protein phosphatase 2 (PP2A) and PHLPP1/2, which dephosphorylate AKT. The activation of the TGF-β/MAPK pathway can be associated with the repression of the transcription of PTEN and a secondary increase in AKT signaling [101].

As with several other pathways, post-translational modification, including ubiquitination and SUMOylation can promote degradation of AKT, maintaining chronic regulation of substrate availability for PIP_3_ [102].

### 5.2. PI3K/AKT and VSMC

The net effect of activation of PI3K/AKT in VSMC is increased proliferation with a synthetic phenotype. In vitro and in vivo murine studies demonstrate that AKT controls VSMC proliferation at least partly by regulating p21 levels and influencing G1/S exit in the cell cycle [103]. At the same time, AKT can inhibit VSMC apoptosis by phosphorylation of proapoptotic proteins such as Bad, caspase 9, glycogen synthase kinase-3 (GSK3), and forkhead class O transcription factor 3a (FOXO3a) [104,105]. In vitro studies of human coronary artery VSMC show that FOXO3a activity inhibits VSMC proliferation. Insulin-like growth factor 1 (IGF-1) stimulates phosphorylation of FOXO3a via MEK1/2 and/or PI3K-dependent signaling pathways, with the subsequent loss of FOXO3a inhibition of cell proliferation [106].

**Figure 5:**
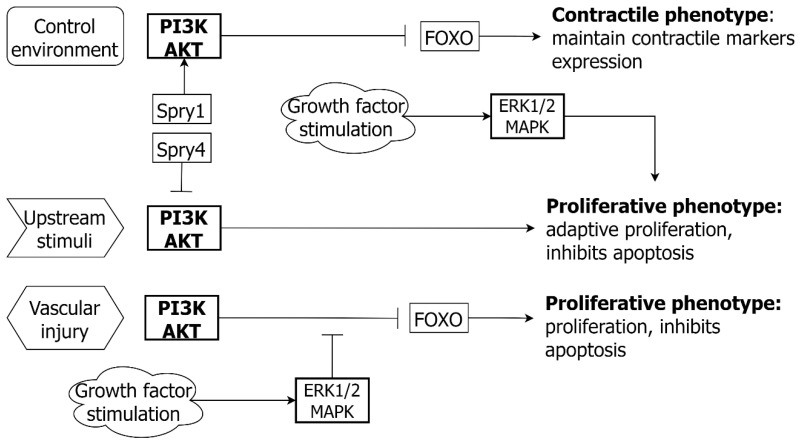
Summary of the mechanisms of PI3K/AKT signaling pathway in VSMC. In the basal state, AKT inhibition of FOXO contributes to maintenance of contractile phenotype. In response to growth factors or vascular injury, upregulated AKT signaling is associated with VSMC proliferation.

In cultured human aortic VSMC, AKT signaling can promote cell proliferation and differentiation. The maintenance of cell differentiation, with contractile phenotype markers, is antagonized by MAPK/ERK (Figure 5). There is a further level of the regulation of AKT signaling in VSMC. The feedback regulator SPRY1 promotes AKT activation, whilst SPRY4 antagonizes AKT with subsequent suppression of cell differentiation [107]. Finally, PI3K/AKT also upregulates anti-apoptotic/suppressor genes, e.g., nuclear factor-κB and murine double minute-2 (MDM2) [108].

In disease states, such as atherosclerosis, PI3/AKT signaling can mediate VSMC responses [109,110]. In murine studies of atheroma, AKT1 activation inhibits VSMC apoptosis during atherogenesis. Multiple FOXO3a-regulated genes are involved in VSMC apoptosis, including apoptotic protease activating factor 1, which is known to be increased in human atherosclerosis, but reduced by AKT1 activity in vivo [108]. The importance of the PI3K/AKT inhibition of FOXO3 is underscored by findings that FOXO3a activation contributes to VSMC apoptosis, ECM degradation and vascular remodeling by promoting transcription of MMP genes with an associated downregulation of tissue inhibitors of MMPs (TIMPs) [111,112].

### 5.3. PI3K/AKT and EC

**Figure 6:**
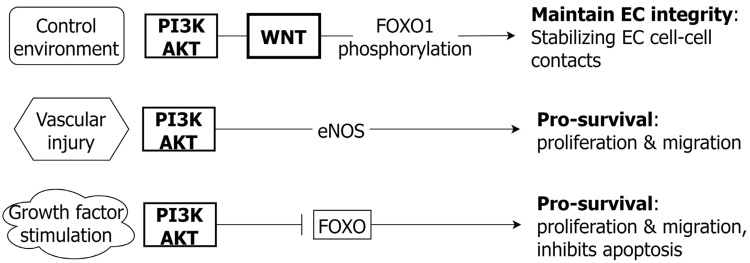
Summary of the mechanisms of PI3K/AKT signaling pathway in endothelial cells. In the basal state, AKT signaling is associated with stability of EC and cell junctions. In response to growth factors, or vascular injury, upregulated AKT activity is associated with EC proliferation.

The activation of the PI3K/AKT pathway promotes EC survival, proliferation, and migration (Figure 6). The PI3K/AKT pathway also promotes NO availability via the increased expression of endothelial nitric oxide synthase (eNOS) when challenged with oxidative stress [110]. As in VSMC, the inhibitory role of PI3K/AKT on downstream FOXO3 promotes cell survival, proliferation, and migration by inhibiting FOXO3a induced EC apoptosis [113,114]. In addition, interaction between PI3K/AKT and the WNT pathway (β-catenin) has a role in EC homeostasis by stabilizing EC cell-cell contacts, via endothelial cadherin and FOXO1 phosphorylation [115].

## 6. NOTCH Signaling

The NOTCH pathway has key roles in determination of cellular architecture, through cell-cell contact signaling, and in the response of cells to the microenvironment, including extracellular matrix characteristics and hemodynamic forces [116]. Unlike other pathways, both ligand and receptor are membrane-bound. Other unique features are that intracellular signaling is a function of cleavage of the receptor, by ADAM17 and gamma-secretase, following ligand binding, yielding the Notch Intracellular Domain (NICD). The NICD translocates to the nucleus, without any intermediate cascade, and binds with members of the CSL family of DNA binding proteins, notably RBPJ. In the absence of NOTCH signaling, CSL functions as a transcriptional repressor, becoming an activator after NICD binding [117]. The pattern of subsequent gene transcription is then regulated by a complex interplay of CSL with a range of cofactors (e.g., MAML, p300). The targets of NOTCH signaling are themselves transcription factors, most notably the basic helix-loop-helix (bHLH) factors, HES, HEY and HERP, which normally repress the transcription of genes encoding proteins involved in tissue differentiation. Other tissue and context specific transcription targets include *EGFR*, *CCND1* and *c-myc* [118].

The NOTCH signaling family includes four different receptors (NOTCH1-4) and different ligands (Dll1, Dll3, Dll4, Jagged1, Jagged2), of which Dll3 is a negative regulator of NOTCH signaling [119]. Aortic VSMC predominantly express NOTCH1-3 and EC express NOTCH 4, Jagged1 and Dll4, providing a mechanism for EC-VSMC communication [120]. Non-canonical NOTCH signaling also occurs via several mechanisms. Endocytosed receptors can be cleaved by ADAM17/secretase in the absence of ligand binding. The NICD can interact with elements of TGF-β, AKT and WNT pathways in both the cytoplasm and nucleus [121]. Several excellent overviews of NOTCH signaling are available [118,119,122].

Disorders associated with mutations in NOTCH genes are uncommon, likely reflecting lethal effects on embryonic development. The CADASIL syndrome, consequent on *NOTCH3* mutations, is a disorder of cerebral arteriolar development and a cause of early stroke. The Alagille syndrome, consequent upon mutations in *JAG1* or *NOTCH2*, is associated with multi-organ developmental abnormalities, especially the liver.

### 6.1. Regulation of NOTCH Signaling

A fundamental feature for NOTCH is the availability and distribution of the membrane-bound ligands and receptors, which reflects balance between synthesis and degradation of each. Other pathways can influence ligand synthesis, with stimulation with TGF-β being associated with an increased expression of Jagged1 [123]. The ubiquitination, endocytosis and recycling/degradation of both ligands and receptors is at the core of determining their availability at the cell surface [124]. In order to avoid inappropriate signaling, and in the absence of direct antagonists, the proteasomal degradation of NICD in the cytoplasm is another major regulatory feature. Within the nucleus, the specificity of signal transduction is regulated by the transcriptional co-factors binding with CSL/RBPJ [125]. Tissue specificity is a key element for NOTCH regulation and NOTCH signaling can result in different outcomes according to tissue type. One element conferring specificity is variation of transcriptional targets, even within the same gene family and differences in enhancers. Another element is variation in transcriptional co-factors between tissues [118].

The cellular microenvironment, notably the ECM, can also influence NOTCH signaling. Several ECM proteins have been described as interacting directly with the NOTCH receptor, including MAGP-2, which can increase NOTCH signaling by binding to NOTCH1 and Jagged-1 [126] or through integrin binding in ECs [127]. Concordant effects on NOTCH signaling in ECs have been shown for EGFL7 (epidermal growth factor like domain multiple 7), which also inhibits PDGF-induced VSMC migration and promotes EC adhesion [127]. Both thrombospondin-2 and syndecan-2 can promote NOTCH3-Jagged1 signaling [128,129], whilst collagen type IV inhibits NOTCH3-Jagged1 signaling [130]. Other ECM proteins, e.g., fibulins, can influence expression of NOTCH receptors and ligands [131]. 

### 6.2. NOTCH and VSMC

**Figure 7:**
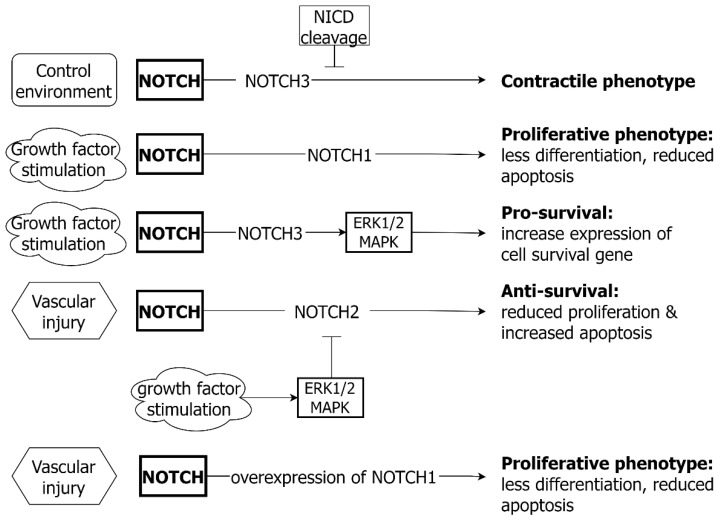
Summary of the mechanisms of NOTCH signaling pathways in VSMC. While NOTCH3 signaling promotes the contractile phenotype, stimulation of other NOTCH isoforms results in proliferative signaling and either cell survival or apoptosis depending on the circumstances.

As with the WNT pathway, NOTCH signaling has a key role in vascular development [132] and also contributes to modulation of VSMC phenotype and vascular response to injury [133,134]. NOTCH signaling from EC influences the differentiation of adjacent VSMC [135]. The activation of NOTCH signaling is associated with VSMC proliferation, differentiation and death, according to context [136]. Different NOTCH receptor-ligand binding interactions yield different outcomes for VSMC survival and proliferation (Figure 7). The constitutive expression of NOTCH1 and NOTCH3 in cultured rat VSMC is associated with cell growth and reduced apoptosis [137].

Under growth factor stimulation (PDGF-B), NOTCH3 expression is elevated in cultured human aortic VSMC, with subsequent MAPK/ERK phosphorylation and the increased expression of cell survival genes [120]. Concordantly, when NOTCH3 is suppressed in aortic VSMC, increased apoptosis is observed [138]. In contrast, the expression of NOTCH2 drives reduced proliferation and increased apoptosis of VSMC [139], which effects are antagonised by the PDGF-B stimulation of the MAPK/ERK pathway. Therefore, MAPK/ERK may function as an intermediary, balancing NOTCH2 and NOTCH3 effects on VSMC survival [120].

Different NOTCH receptor-ligand binding drives different outcomes for VSMC differentiation and phenotype [132]. In cell culture, the JAG-1 stimulation of NOTCH3 results in a contractile phenotype [140,141,142], which process is blocked by the inhibition of NICD cleavage. The opposite effects are observed in aortic VSMC with the overexpression of NOTCH1, which results in the loss of expression of contractile markers and a less differentiated phenotype [143], which in turn has been implicated in the proliferative response to vascular injury [144]. In a model of vascular injury, NOTCH1, NOTCH3, HAY1, HAY2 and JAG-1 all exhibit an initial decrease in expression followed by an increased expression. In aortic VSMC, the inhibition of NOTCH signaling promotes MMP2 and MMP9 expression, contributing to ECM degradation [145].

### 6.3. NOTCH and EC

**Figure 8:**
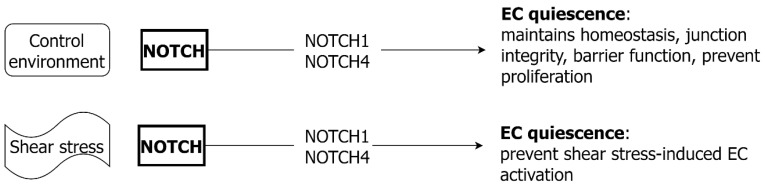
Summary of the mechanisms of NOTCH signaling pathways in EC. Generally, the role of NOTCH signaling in EC is to maintain the quiescent phenotype, particularly suppress shear stress-induced activation.

NOTCH signaling in EC serves to maintain vascular homeostasis and endothelial junction integrity (Figure 8). Most ECs are in a quiescent state, with NOTCH signaling suppressing EC response to growth factors such as VEGF [146]. NOTCH1 is sensitive to shear stress and serves a mechanosensory function in the vasculature [147]. NOTCH signaling is responsible for stabilizing cell junctions and preventing excessive EC proliferation induced by shear stress [147,148]. The binding of NOTCH1 and NOTCH4 receptors by Jagged1 or Dll4 results in reduced EC proliferation [149] and the inhibition of Dll4 binding results in EC proliferation [150]. Whilst EC-released NOTCH ligands binding to VSMC NOTCH receptors can regulate VSMC proliferation, a reverse signaling pathway also exists [141]. Intact VSMC NOTCH signaling via JAG-1 is needed for efficient EC NOTCH1 receptor activation and EC proliferation [151]. Non-canonical NOTCH signaling also occurs in the EC in response to shear stress, through the membrane bound receptor without NICD cleavage. This mechanism has also been reported to promote endothelial integrity and barrier function [152].

## 7. Angiotensin Signaling

The renin-angiotensin system includes a number of ligands, of which angiotensin II (ANG II) is the best known. Other ligands are angiotensin 1-7 (ANG 1-7) and angiotensin 1-12 (ANG 1-12). Signaling is via ligand binding to GPCR cell surface receptors, including AT1R, AT2R and MAS, through which ANG 1-7 exerts effects antagonistic to ANG II /AT1R signaling. Following ANGII/AT1R binding, complex cascades of intracellular signaling, involving G proteins, serine/threonine and tyrosine kinases are activated. Other effectors of ANGII/AT1R binding include downstream actions through ROS (via NADPH oxidase) and PLA/PLC. Indirect effects of ANGII/ATIR binding include the upregulation of growth factor signaling, with the transactivation of EGFR, PDGFR and IGF-1R in VSMC. The vascular consequences of ANG II/AT1R signaling include arterial vasoconstriction, vascular fibrosis and remodeling and altered endothelial function. In contrast, ANG II/AT2R signaling results in vasodilatation, reduced cell proliferation and reduced fibrosis in both EC and VSMC [153]. Although AT2R expression is reduced after birth, AT2R remain detectable in the adult cardiovascular system. A major mechanism of action for ANGII/AT2R is through serine/threonine or tyrosine phosphatases, thereby directly antagonising ANGII/AT1R signaling. For further information on ANGII signaling in the cardiovascular system, several recent reviews are available [154,155,156].

### 7.1. Regulation of ANG II Signaling

Changes in receptor density are a key regulatory feature of ANG II signaling. Firstly, ANGII itself downregulates the expression of AT1R, with chronic exposure [157]. In addition, multiple hormones and growth factors can upregulate (insulin, progesterone) or downregulate (estrogen, epidermal growth factor, platelet-derived growth factor) AT1R expression [154]. As with TGF-β receptors, the endocytosis, recycling and degradation of AT1R provides additional regulation [158]. Other mechanisms influencing AT1R density include induction of iNOS resulting in the suppression of NF-*K*B [159] and post-translational modifications, e.g., tissue transglutaminase action on AT1R results in the inhibition of ubiquitination and degradation of the receptor.

### 7.2. Angiotensin II and VSMC

The principal effects of chronic ANGII/AT1R on VSMC include increased contractility, hypertrophy, fibrosis and cell migration [160]. The ECM is also affected with an increased turnover of glycosaminoglycans, matrix metalloproteinase activation and the degradation of structural elements. The overall consequence is vascular remodeling (arteriosclerosis) with stiffer arteries, hypertension and accelerated vascular disease. In VSMC, ANGII/AT1R results in vasoconstriction via the G protein-mediated activation of myosin light chain kinase and the inhibition of myosin light chain phosphatase. Other downstream signaling includes various serine/threonine and tyrosine protein kinases e.g., MAPK and AKT. In VSMC, ANGII stimulation results in upregulation of systems involved in protein synthesis and myocyte contraction, whilst also upregulating synthesis of ECM proteins and signaling ligands, including TGF-β [161].

In both VSMC and EC, ANGII/AT1R stimulation results in chronic inflammation, through the activation of NF-*K*B and generation of ROS via NADPH oxidase [162]. In addition, ANGII/AT1R can promote VSMC and cardiac myocyte hypertrophy, proliferation and apoptosis, independently of effects on blood pressure and mediated by MAPK/ERK [163]. Whilst ANG II does not induce the proliferation of human aortic VSMCs in vitro, ANG II infusion in mice causes macrophage-dependent complement C1q production, which in turn stimulates the β-catenin pathway and proliferation of VSMC in vivo.

**Figure 9:**
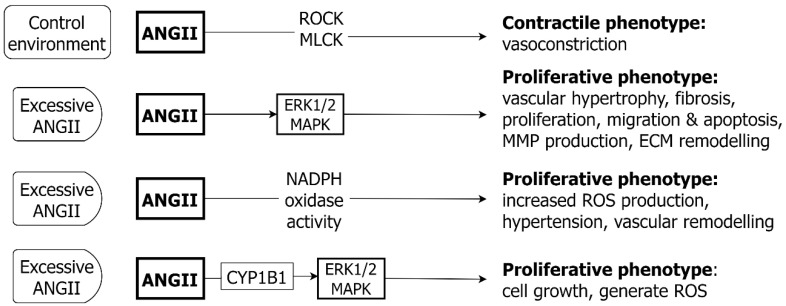
Summary of the mechanisms of ANGII signaling pathways in VSMC. While the immediate effect of ANGII stimulation is to maintain vascular tone, chronic and excessive stimulation of ANGII results in a proliferative phenotype. The generation of ROS results in inflammation that also promotes proliferation.

There are multiple pathways described by which ANGII/AT1R stimulation induces VSMC hypertrophy (Figure 9). Firstly, ANGII/AT1R binding can result in the transactivation of EGFR, with the subsequent activation of ERK1/2 and VSMC hypertrophy. Exposure to ANGII results in phosphorylation of the transmembrane metalloproteinase ADAM17, promoting the activation of EGFR [164]. In turn, EGFR stimulation promotes ERK signaling, with a potential feedback loop through the further promotion of ADAM17 expression [155]. A parallel pathway for ANGII mediated hypertrophy is via JAK2 and ROCK activation [165]. Thirdly, the serine/threonine kinase CamKII can also mediate VSMC hypertrophy in response to ANGII by the activation of MEF2, a transcriptional regulator of genes involved in the hypertrophic response [166]. Other kinases involved include mitogen-activated protein kinase-activated protein kinase 2 (MK2) and PKC-δ [167].

Another mechanism of ANGII-induced VSMC hypertrophy appears to be via ROS, generated by increased NADPH oxidase activity [168]. Several transgenic mouse models exhibit increased ROS production, hypertension and vascular remodeling in response to ANGII, including Nox1^−/−^ mice [1], Sod1^−/−^ mice [169] and mice with an inducible Nox4 deletion [170]. ANGII can induce VSMC growth through MEK/ERK1/2 via a cytochrome P450 1B1 (CYP1B1) gene-dependent pathway, with the further generation of ROS [171].

Other detrimental effects of increased ANGII/AT1R signaling are the increased expression and release of MMPs from VSMC, contributing to vascular remodeling [172]. Abnormal vascular stiffness, with increased collagen and degraded ECM proteoglycans, are observed in the aorta following chronic ANGII/ATIR stimulation [173]. In models of atherosclerosis, VSMC can migrate and proliferate within the degraded ECM [174], however, this is not a feature of TAA. The magnitude of the effect of ANGII upon ECM stiffness is likely to be mediated by the balance of MMP and TIMP concentrations in the ECM.

### 7.3. Angiotensin II and EC

**Figure 10:**
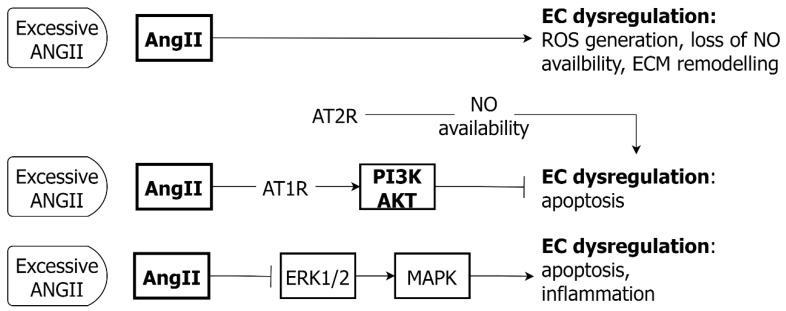
Summary of the mechanisms of ANGII signaling pathways in EC. Excessive ANGII will result in EC dysregulation through the generation of ROS, result in inflammation, ECM remodeling and apoptosis.

Effects of ANGII/AT1R activation in EC include generation of ROS (via NADPH oxidase), which is also associated with loss of NO availability, as NO complexes with superoxide to form peroxynitrite. The ROS can also deplete cofactors for eNOS, further impairing NO synthesis [175]. A pro-inflammatory state for ECs results, with an increased expression of VCAM-1 and altered EC adhesion properties [176]. At the same time, ANGII/AT1R stimulates EC release of matrix metalloproteinases, which contribute to ECM remodeling [177] (Figure 10). Exposure to ANGII influences survival in EC and may promote or inhibit apoptosis, according to context. The inhibition of apoptosis is via AT1R/PI3K/AKT pathway, whilst apoptosis can be induced via AT2R, according to NO availability [178,179]. ANGII signaling is linked to ROS, with the promotion of EC apoptosis by, firstly, the inhibition of ERK1/2 and the activation of MAPK and decrease in Bcl-2 anti-apoptotic protein and, secondly, via JNK with the activation of NF-κB and the induction of inflammation [179,180].

The EC appear to have a role in mediating TAA formation in response to ANGII stimulation. Whilst ANGII directly stimulates VSMC hypertrophy and fibrosis, through multiple signaling mechanisms, TAA formation in the context of dysregulated ANGII signaling requires the participation of EC. Mice with a VSMC-specific AT1R deletion, receiving a sustained ANGII infusion develop TAA, but mice with an EC-specific AT1R deletion do not develop TAA. Thus, EC response to ANGII appears to result in altered VSMC behaviour and eventual TAA [30]. Other evidence for the role of EC comes from experimental findings showing that ANGII induces MMP2 expression with associated TAA, even without growth factor stimulation of VSMC [181].

## 8. Interactions between Pathways

**Figure 11:**
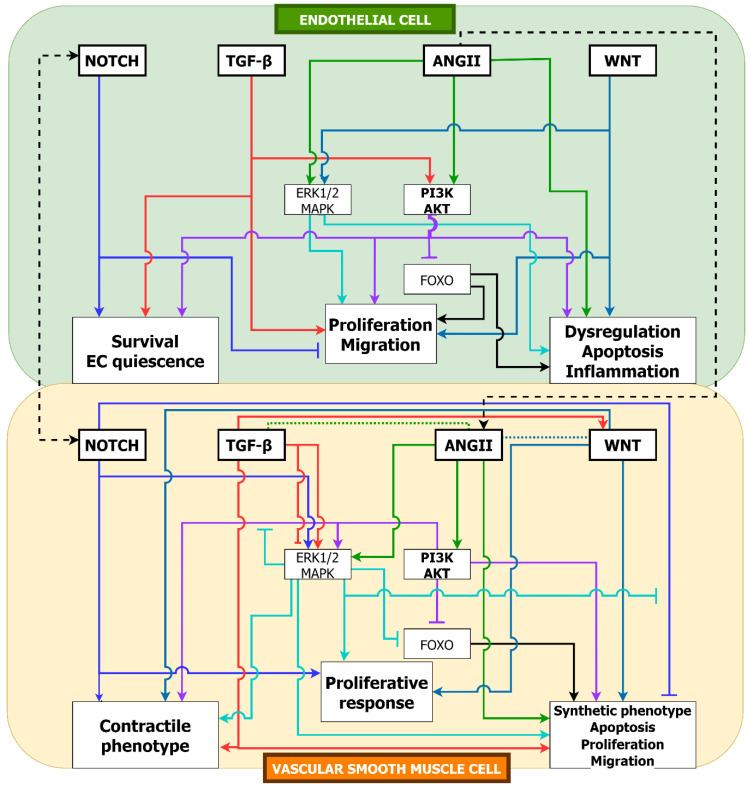
Summary of pathways and direction of activity in promoting different cellular responses in EC and VSMC, via either direct or indirect activation. Arrows indicate activation whereas the stops illustrate inhibition of the pathway/process. Colored dashed lines indicate binding and/or co-signaling. Black dashed line indicates the process involves VSMC-EC interaction. Solid lines correspond different signaling pathways: Red, TGF-β; Teal, WNT; Purple, PI3K/AKT; Blue, NOTCH; Green, ANGII; Light blue, ERK1/2/MAPK and Black, FOXO. This figure illustrates the extend of signaling redundancy. Additionally, the PI3K/AKT and MAPK pathways are often common intermediaries. For details of signaling pathways refer to the text.

An important feature of each of the pathways described is their interactions with others, which can result in both synergistic and antagonistic effects on cell function and phenotype. These interactions play a key role in tissue development and homeostasis and are likely to be similarly significant in the pathogenesis of TAA. Signal redundancy is a feature of many pathways. In consequence, the inhibition of one pathway or sub-pathway might be of only limited efficacy in altering cell fate, due to a common downstream effector receiving signals from another pathway. As shown in Figure 11, PI3K/AKT and MAPK are two common intermediate pathways in VSMC and EC, which can impact cell destiny. Bidirectionality is also a feature of several pathways, in which activation can have alternate effects on VSMC and/or EC phenotype, according to context. As TGF-β, WNT and PI3K/AKT signaling may each exhibit bidirectional outcomes in VSMC and EC, their expression is tightly regulated in the healthy aorta [182].

The TGF-β/BMP pathway exhibits a broad range of interactions with other signaling pathways, many of which are themselves involved in the pathogenesis of TAA [15,183]. Non-canonical signaling by TGF-β, particularly via MAPKs, ERK 1/2, JNK 1/2, and p38 underlies several signaling pathway interactions [98,184]. Growth factors, e.g., EGF, FGF and PDGFR, upon binding to membrane receptor tyrosine kinases (RTKs), stimulate Ras activation, with the upregulation of PI3K/AKT activity, as well as the MAPKs. Both ERK1/2 and GSK3-β can phosphorylate the linker region of SMAD3, thereby inhibiting its transcriptional activity [185,186].

Similar outcomes are observed with ERK1/2 and GSK3-β phosphorylation of SMAD 1/5, with subsequent degradation by the E3 ubiquitin ligase SMURF1, thereby inhibiting BMP/SMAD signaling [187]. The MAPKs can also impact upon TGF-β/BMP/SMAD signaling via the phosphorylation of target transcription factors c-Jun, c-Fos, AP-1 and ATF. As a result, growth factors can antagonize the apoptotic outcomes of TGF-β/BMP/SMAD signaling, whilst potentiating the proliferative and inflammatory outcomes of non-canonical TGF-β/BMP/MAPK signaling. Feedback loops may occur, as TGF-β/SMAD signaling can itself stimulate growth factor expression.

Tissue growth regulation depends, to a large extent, upon interactions between TGF-β/BMP and WNT signaling and multiple experimental studies have described interactions between the pathways. Studies in a variety of tissues have shown that each pathway can contribute towards regulation of ligand synthesis for the other. In cultured VSMC, TGF-β/SMAD3 stimulation is associated with the upregulation of multiple WNT ligands, increased β-catenin and enhanced VSMC proliferation [57]. Similarly, BMP2/4 can influence WNT7a/8 synthesis, whilst WNT/β-catenin can also stimulate the expression of BMP antagonists [188].

In the cytoplasm, multiple cross-interactions influence pathway activity. Thus, binding of AXIN-1 to SMAD3 can accelerate the phosphorylation of SMAD3 by GSK3-β, with subsequent ubiquitination/degradation, with this process being independent of WNT-FZD binding [189]. Other studies have shown that binding of AXIN-1 to SMAD3 is associated with an enhanced phosphorylation of SMAD3 by TGFBR1 kinase and promotion of TGF-β/SMAD signaling [190]. These opposite outcomes of AXIN-1/SMAD3 binding are likely a consequence of the phosphorylation of different specific sites on SMAD3 and also subcellular localization (caveolar vesicles vs. free cytoplasmic). Conversely, SMAD7 can bind to β-catenin, promoting its degradation via SMURF2 [191]. The balance of activity between the pathways is complicated by findings that SMAD7 is itself a target for AXIN-1 binding, resulting in ubiquitination/degradation of SMAD7, with a subsequent increase in TGF-β/SMAD3 signaling [192,193]. In the other direction, SMURF1/2 can influence WNT/β-catenin signaling by targeting AXIN-1 [194]. In the nucleus the SMAD proteins and β-catenin/TCF/LEF regulate the transcription of many shared genes, with both synergistic and antagonistic outcomes [195]. The transcriptional interactions of TGF-β/BMP and LEF/TCF appear to be ligand- and context-dependent.

Tissue fibrosis, consequent upon TGF-β signaling, may require the activation of canonical WNT signaling, and the antagonism of WNT through SOST and DKK1 is associated with reduced fibrotic response to TGF-β [196,197]. In pulmonary VSMC, the effect of BMP upon VSMC (increased motility and reduced proliferation) is dependent upon the tandem activation of the canonical and non-canonical WNT pathways [198].

There are multiple interactions between ANGII and other signaling pathways. In VSMC ANGII regulates TGF-ß expression and the latter mediates ANGII induced fibrosis and ECM remodeling [199,200]. Experimental studies confirm that both TGF-β and downstream SMAD3 are required for ANGII-induced vascular fibrosis [201]. Furthermore, ANGII-induced TNF facilitates cardiac interstitial and perivascular fibrosis through CTGF, and TGF-β [202]. In the absence of TGF-β stimulation, AT1R expression in VSMC is increased, and conversely AT1R expression is suppressed with TGF-β stimulation, with reduction in VSMC proliferation [203]. Chronic ANGII infusion in rats promotes MAPK activation, consequent upon AT1R binding and independent of TGF-ß receptor binding. The activation of MAPK is blocked by the AT1R antagonist, losartan [204].

In cardiac myocytes, WNT/ß-catenin signaling antagonizes ANGII-induced myocyte hypertrophy [205], whilst ANGII induces the expression of the WNT/ß-catenin regulated WISP1 (WNT-induced secreted protein-1), a TCF/LEF target for cardiac hypertrophy [206]. In VSMC, ANGII stimulation appears to increase nuclear ß-catenin levels and stabilize vascular remodeling. In human VSMC, ANGII/AT1R binding can stimulate NOTCH signaling via enhanced nuclear translocation of NICD, whilst the inhibition of NICD release from the NOTCH receptor antagonizes ANGII-induced VSMC hypertrophy [207]. Inhibition of NOTCH3-NICD signaling attenuates ANGII/ATIR- mediated vasoconstriction, through reduced expression of MLCK, which regulates VSMC contractility [208].

Interactions between NOTCH and TGF-β pathways may be either synergistic or antagonistic, depending upon context, in particular cell contact [209]. In VSMC and EC, TGF-β and NOTCH signals can be integrated. Signaling by TGF-β can induce the expression of NOTCH-related transcription factors HES1, HEY1 and JAG1, via a SMAD3/NCID interaction [210]. At the same time, NOTCH can promote the expression of some TGF-β induced genes, whilst reducing the expression of others [211]. There is also evidence that NOTCH can have different effects on the expression of SMAD3 vs. other SMADs.

At the nuclear level, SMAD3 and NICD can cooperatively regulate gene expression. Crosstalk between the NOTCH and TGF-β pathways appears mediated by the common effector CBF1, leading to increased pSMAD2/3 promoter binding when NOTCH is added to TGF-β, but not vice versa. Antagonistic effects have been reported in EC after BMP stimulation [212]. In absence of cell contact, BMP stimulation promotes EC migration via upregulation of ID1. In the presence of cell contact, NOTCH signaling is triggered with the upregulation of HERP2 and the suppression of cell migration, with the degradation of ID1 promoted by HERP2. Further antagonistic effects of NOTCH upon TGF-β/BMP signaling are observed when NICD sequesters either SMAD2/3/4 or the transcriptional cofactor p300 [213,214].

In cultured aortic VSMC, both NOTCH and TGF-β stimulation induce a contractile phenotype, with combined stimulation resulting in greater expression of contractile proteins than either pathway alone [140]. The expression of JAG1 also supports TGF-β signaling promoting cytostasis [215], whilst it appears that NOTCH is cooperative with TGF-β in the process of tissue fibrosis [216,217].

There can also be both synergistic and antagonistic actions of WNT and NOTCH [218,219]. Non-canonical Ca^++^ dependent WNT signaling can influence NOTCH1 signaling [220]. In EC, β-catenin increases DLL4 transcription, with associated increase in NOTCH signaling resulting in altered vascular remodeling [221]. These interactions are dependent upon stages of vascular development. Conversely, NOTCH can restrict WNT canonical signaling by promoting the endocytic sequestration of β-catenin at the cell membrane and also by limiting the β-catenin induced transcription of WNT target genes [222]. Crosstalk between WNT and NOTCH pathways may also be mediated by GSK3-β, normally inactivated by WNT, which can phosphorylate NICD, with subsequent ubiquitination and degradation, to inhibit signaling [9]. Direct interactions between NOTCH NICD and LEF, or between NICD and β-catenin, independent of canonical signaling in either pathway, yield transcriptional complexes regulating specific gene sets [223,224].

The PI3K/AKT pathway fulfills an integrative function for multiple signaling pathways. Numerous interactions between TGF-β/SMAD and PI3K/AKT are described [15], which can be synergistic or antagonistic according to cell context. In response to EGF, IGF and IL6 binding to RTKs, PI3K/AKT can inhibit TGF-β/SMAD signaling. Evidence exists for several mechanisms of interaction, including the antagonism of SMAD3 activation by TGFBRII and the phosphorylation of SMAD3 with impairment of its nuclear translocation [225,226]. In addition, AKT can mediate the phosphorylation of key transcriptional co-factors of SMAD3, such as FOXO [227]. The balance between TGF-β/SMAD and PI3K/AKT signaling is evident in observations that AKT can deubiquitinate and inhibit degradation of the TGFBR1 receptor [15,228], thereby enhancing TGF-β/SMAD3 signaling, whilst AKT has the potential for the direct phosphorylation of SMAD3, independent of TGF-β/TGFBR binding [229]. The different outcomes of AKT mediated phosphorylation of SMAD3 appear to reflect the molecular location of phosphorylation, illustrating the precision of interactions between the two pathways. There is also likely feedback regulation between the pathways, as TGF-β/MAPK stimulation can result in increased AKT activity.

There is evidence for functionality of these interactions in the aorta. In cultured human aortic VSMC, TGF-β stimulation resulted in PI3K/AKT activation and an increased expression of the inhibitory transcriptional regulator ID2, with consequent reduced expression of VSMC contractile marker proteins, SM-22α and α-SMA and an increased expression of the synthetic phenotype protein osteopontin. These effects could be reversed by the use of a PI3K/AKT-specific inhibitor [230,231]. Whilst TGF-β signaling is usually associated with the repression of VSMC proliferation, arterial injury is associated with the upregulation of TGF-β and intimal EC hyperplasia. Increased TGF-β stimulation, with elevated SMAD3 levels, results in the phosphorylation of AKT, with pAKT then driving cell proliferation [45]. The extent of AKT phosphorylation appears dependent upon SMAD3 levels.

The PI3K/AKT pathway also has a role in regulating the activity of WNT signaling. In summary, WNT signaling activates AKT, which in association with Dishevelled, enhances the phosphorylation of GSK3-β in the AXIN complex. The phosphorylated GSK3-β cannot modify β-catenin, so that it accumulates in the cytosol, translocates to the nucleus and combines with LEF/TCF co-factors to upregulate WNT-dependent gene expression [232].

Another major interactor with PI3K/AKT is the angiotensin pathway. In cultured VSMC, ANGII/AT1R binding activates the PI3K/AKT pathway with subsequent MAPK activation and VSMC proliferation [233]. Thus, PI3K/AKT can act as an effector of vascular growth in response to ANGII. Interestingly, the overexpression of AKT1 in cultured cells results in the suppression of cell proliferation, which effect appears mediated by the inhibition of nuclear translocation of ERK1/2 [234].

### Summary of Pathway Interactions

**Figure 12:**
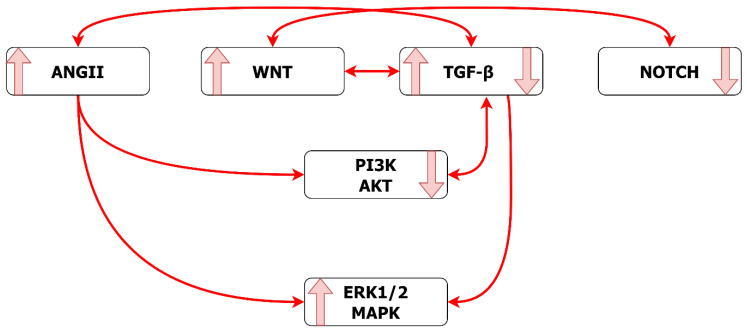
Summary of pathway crosstalk in TAA. Red lines indicate that there is evidence of the interaction in TAA. The large pink arrows indicate increased and/or decreased activity of the pathway in TAA.

Interactions of multiple pathways, influencing outcomes for EC and VSMC, illustrate some key features, relevant to understanding the pathogenesis of TAA (Figure 11 and Figure 12 and Table 1). Whilst the activation of a pathway may exert independent signaling, with direct effects on VSMC or EC, the same pathway may serve as an upstream stimulus for the activation/inhibition of another downstream pathway, with indirect effect on cell destiny. One pathway may serve to integrate competing stimuli from multiple upstream pathways, resulting in a balanced effect on cellular outcomes. As summarized in Table 1, TGF-β, WNT and PI3K/AKT signaling have both direct and indirect impacts on VSMC and EC also demonstrated bidirectionalities. In contrast, NOTCH demonstrated direct and indirect impact on VSMC but not on EC, with no bidirectionality shown in EC. ANGII exhibits direct and indirect impacts on both VSMC and EC but only promotes detrimental responses in VSMC. However, ANGII in EC demonstrated more varieties, further illustrating that the EC-ANGII receptor might be the primary receptor for ANGII signaling. Balanced interactions between pathways, including intra- and inter-pathway feedback loops, underpin normal tissue growth and homeostasis. In consequence, disease processes, including TAA formation, are likely to involve disturbances in multiple pathways.

## 9. Role of Signaling Pathways in Pathogenesis of TAA

### 9.1. TGF-β and TAA

Interest in TGF-β signaling in TAA arises from observations that mutations in the *FBN1* gene cause MFS, the commonest form of syndromic TAA and fibrillin-1 is implicated in TGF-β tissue homeostasis [235]. It has been proposed that TAA in MFS results from the failure of TGF-β sequestration in the ECM. Mutations in genes encoding TGF-β cell surface receptors (*TGFBR1*, *TGFBR2)* cause LDS, characterized by systemic arterial aneurysms [236]. The related aneurysm-osteoarthritis syndrome is consequent upon mutations in the *SMAD3* gene [237,238]. Following the clinical description of LDS, several studies have documented a role for TGF-β in the pathogenesis of TAA [13,239,240]. Mice with either TGFBR1 or TGFBR2 deficiency develop TAA with evidence of reduced SMAD3 signaling, but increased ERK1/2 activity, consistent with the role of MAPK/ERK in mediating VSMC phenotype switching [241,242].

The inhibition of TGF-β by neutralizing antibody has been reported to attenuate TAA formation, however effects appear to be time and dose-dependent. Thus, the administration of TGF-β antibody before aneurysm formation can attenuate aortic growth but increases the risk of rupture after TAA formation [243]. Furthermore, with high-dose TGF-β antibody administration, mice receiving ANGII infusion had significantly increased risk of aortic rupture [240].

Mice with fibrillin-1 deficiency show an increased expression of ERK1/2 and MAPK kinase-1 (MEK1), however after treatment with a TGF-β-neutralizing antibody or losartan, ERK1/2 expression was significantly decreased. Additionally, both a MEK1/2 inhibitor and a JNK inhibitor decreased aortic growth in the aortic root and ascending aorta [184]. These findings suggest that noncanonical TGF-β signaling and interaction with MAPK/ERK signaling contributes to TAA.

In TAA associated with BAV, altered aortic flow patterns consequent upon the abnormal aortic valve, may result in disordered EC signal transduction [30]. There is, however, conflicting evidence regarding the role of TGF-β in pathogenesis of TAA in BAV. Aortic VSMCs from BAV and tricuspid aortic valve (TAV) aneurysms exhibit different gene expression patterns after TGF-β stimulation [244], with evidence for the downregulation of the response to TGF-β in BAV, which may reflect higher levels of the TGF-β binding protein LTBP4. In contrast, proteomic studies have found evidence of increased TGF-β signaling activity in BAV TAA [245]. As with MFS, circulating blood TGF-β levels have been reported to be elevated in BAV TAA [246], although the exact relationship between circulating TGF-β and TAA formation is not established.

Recent murine experimental evidence shows that crosstalk between EC and VSMC mediates TAA formation when TGF-β signaling is disrupted by mutations in the VSMC *Tgfbr2* receptor genes [50]. Despite the fact that TGF-β signaling stimulates contractile protein expression in VSMC, no reduction in VSMC contractile proteins was observed in *Tgfbr2^+/−^* mice. Instead, EC dysfunction with reduced eNOS activity and associated aortic VSMC hypercontractility were observed, the mechanisms of which was not defined.

Both clinical associations and experimental evidence support a role for TGF-β in TAA pathogenesis in humans, however, there remains uncertainty about the precise features of TGF-β in different genetic TAA [247]. It appears likely that the role of TGF-β signaling may differ between types of TAA and also be dependent upon the stage of aneurysm development.

### 9.2. WNT and TAA

During the last decade, evidence has accumulated to support a role for both canonical and non-canonical WNT signaling in the pathogenesis of CVD, including atherosclerosis, valve disease and vascular fibrosis. Changes in WNT signaling have been observed in both VSMC and EC, in models of atherosclerotic disease and vascular injury. Intimal hyperplasia, with VSMC proliferation, after vascular injury is mediated in part by WNT4/β-catenin signaling [86]. Non-canonical WNT signaling has been shown to influence VSMC phenotype and endothelial cell function [71]. Non-canonical signaling, triggered by WNT5A, has been associated with the induction of reactive oxygen species (ROS) and a pro-inflammatory state in the endothelium and VSMC phenotype switch towards a synthetic state.

The WNT/β-catenin signaling pathway is a regulator of endocardial cushion maturation and valve leaflet stratification [248] and has been reported to contribute to myxomatous human mitral valve disease, as occurs in MFS [249]. Experimental studies show that WNT/β-catenin signaling is activated specifically in valve interstitial cells located in the thickened/diseased mitral valve of a murine model of MFS [250]. Aortic valve calcification (which is common in BAV) has also been shown to be associated with increased WNT/β-catenin signaling [251]. In human calcific aortic valves, both WNT5a and WNT11 through non-canonical signaling have been associated with severity of calcification [252].

The MFS is characterized by the disordered assembly of fibrillin microfibrils in the ECM. The actual mechanism of disorder remains to be fully defined, however, WNT signaling appears to regulate fibrillin assembly. In the dermis, WNT has been shown to influence fibrillin deposition in the ECM, in concert with TGF-β [253]. Similar findings have been reported in MFS [254]. Both TGF-β and WNT signaling have also been implicated in pathogenesis of cardiac fibrosis. In cardiac fibroblasts, WNT/β-catenin signaling promotes the TGF-β-mediated fibroblast-to-myofibroblast transition by enhancing interleukin-11 production [255].

In human non-BAV TAA, increased canonical WNT/β-catenin signaling in EC appears to be associated with reduced baseline NOTCH signaling and abnormal EC responses to shear stress [256]. The TGF-β signaling ligand (BMP2) can activate WNT/β-catenin signaling and increased BMP2 and WNT/β-catenin effectors (TCF7L2, DKK1) are observed in ECs from TAA.

The WNT/β-catenin/TCF-LEF pathway effector, TCF7L2 is known to influence VSMC phenotype and survival [257]. A recent genome-wide association study in non-syndromal TAA identified a locus in the third intron of the gene coding for TCF7L2 [258], with the TAA risk allele being associated with higher expression of TCF7L2 in the aorta. In isolated human arterial smooth muscle cells, the increased expression of TCF7L2 was associated with reduced BCL2 and altered BAX/BCL2 ratios consistent with a pro-apoptotic effect of TCF7L2. In human TAA tissue, reduced BCL2 and increased BAX/BCL2 ratio has also been reported [259]. These data indicate that the WNT signaling pathway is a likely candidate for mediating the pathogenesis of both valvular disease and TAA formation in several genetic aortopathies.

### 9.3. PI3K/AKT and TAA

There is both in vitro and in vivo data to support a role for abnormal PI3K/AKT signaling in the pathogenesis of TAA. In abdominal aortic aneurysm, a role for PI3K/AKT signaling has been well recognized, consistent with the relationship between PI3K/AKT and the inflammatory response [260,261,262]. Less information has been available about PI3K/AKT in TAA. There does appear to be a role for the PI3K/AKT pathway in integrating feedback between TGF-ß, ANGII and growth factors (PDGF, IGF) in TAA [263]. In ascending TAA associated with BAV, the reduced expression of AKT, p-AKT, p-ERK1/2 and extracellular superoxide dismutase (SOD3) has been observed, whereas changes in TAV associated TAA were minimal [264]. The expression of SOD3 is associated with activation of PI3K/AKT and ERK1/2 pathways, with reduced VSMC apoptosis. In contrast, if AKT expression is reduced, the apoptosis of VSMC is increased and at the same time MMP-9 levels are increased, with associated ECM degradation.

Different findings have been reported by Hirata et al. [265]. In human BAV aortopathy, apparent activation of AKT signaling (increased pAKT) was observed in the media of the dilated ascending aorta, with the identified stimulus being fibroblast growth factor (FGF). The investigators also noted that NOTCH1 can repress PI3K/AKT activity and given association of *NOTCH1* variants with BAV, this may be an alternate stimulus to AKT signaling.

In a murine model of MFS, the impaired phosphorylation of both eNOS and AKT has been observed in the thoracic aorta, but not abdominal aorta, and endothelial vasoregulation is impaired [266]. Although the AT1R blocker losartan partly restores endothelial function, impaired eNOS and AKT signaling persists [267]. In cultured human VSMC extracted from TAA, the lncRNA MIAT is increased and this lncRNA has been shown to inhibit AKT signaling, with associated reduction in VSMC viability [268].

Disturbances of PI3K/AKT signaling appear to be a feature of TAA in both experimental and human studies. Given the intermediate role of PI3K/AKT downstream of several receptor/ligand systems, discovery of the altered stimuli influencing pathway activity may identify new treatment interventions.

### 9.4. NOTCH and TAA

Whilst the role of NOTCH in aortic valve development and BAV has been a focus of many investigations [269,270], there is less information about the role NOTCH signaling in maintenance of aortic architecture or development of TAA [271]. In clinical studies, mutations in the NOTCH1 gene have been associated with BAV and TAA [269,272,273]. Follow-up studies in *Notch^+/^^−^* mice have also suggested a role for NOTCH in the development of TAA [274]. 

Pathology studies show that BAV TAA is associated with increased aortic VSMC apoptosis (even before overt aneurysmal dilatation) [275]. A reduction in NOTCH1 and NICD has been described in VSMC in TAA [276] and abnormal patterns of NOTCH regulatory patterns also occur in TAA, with reduced NOTCH1 activity and increased VSMC apoptosis [277]. It is possible that NOTCH2 expression may be increased in TAA, further driving VSMC apoptosis.

In vitro study of human EC derived from BAV TAA shows the reduced expression of NOTCH1 and DII4, with attenuated NOTCH1 responses after TGF-ß stimulation compared to normal TAV aorta [278,279]. In a study of human aortic VSMC from TAA, Zou et al. observed features consistent with the downregulation of NOTCH signaling, although opposite features were observed in stem cells and macrophages [276]. The data suggest that impaired NOTCH signaling in VSMCs may contribute to the apoptosis and loss of VSMC in TAA. Contrasting data in non-BAV TAA describes the upregulation of NOTCH signaling proteins [280], illustrating continuing uncertainty about the role of NOTCH signaling in VSMC in TAA.

In a murine model of MFS, Jespersen et al. have recently reported evidence of increased NOTCH3 expression and activation in ascending aortic tissue. The blockade of NOTCH signaling with the *γ*-secretase inhibitor *N*-[*N*-(3,5-difluorophenacetyl)-l-alanyl]-*S*-phenylglycine t-butyl ester (DAPT) attenuated aortic dilatation [281]. There is also evidence that EC NOTCH signaling is abnormal in adult TAA of non-genetic origin [276]. In adult aortic EC from TAA, WNT/ß-catenin activity was increased, but NOTCH1 and DII4 expression were decreased (opposite to the findings in the developing vasculature).

There appears to be a role for NOTCH signaling in mediating VSMC apoptosis in TAA, with the actual outcomes likely dependent upon NOTCH subtype. It is less likely that alterations in NOTCH signaling are a primary cause of TAA, but rather that NOTCH functions as a modulator of the disease process.

### 9.5. Angiotensin II and TAA

The adverse remodeling of the ECM is associated with chronic ANGII/AT1R signaling, with increased collagen deposition and matrix glycosaminoglycan synthesis [282]. In abdominal aortic aneurysm, characterized by atherosclerotic disease and inflammation, ANGII/AT1R signaling plays a prominent role and there is also evidence that ANGII signaling contributes to TAA [283]. The main evidence supporting a role for ANGII in TAA is from various murine models, including EC At1r^−/−^ Ldlr^−/−^ mice, which show characteristic changes of medial necrosis in response to ANGII [284], as do those with VSMC Lrp1^−/−^ after ANGII exposure [285].

Several murine models of genetic TAA have been studied, including Marfan syndrome (*FBN1* mutations), Loeys-Dietz syndrome (*TGFBR1*, *TGFBR2* mutations) and familial TAA associated with *ACTA2* mutations. Each of these syndromes is characterized by TAA at a young age and also by cerebral aneurysms (Loeys-Dietz and ACTA2). In a fibrillin-deficient Marfan mouse model, the loss of ANGII-AT1R signaling in EC is associated with reduced p-ERK1/2 activity and reduced TAA formation, suggesting a role for ANGII-stimulated ERK1/2 activity in pathogenesis of TAA [286]. Other evidence suggests that altering the balance of AT1R and AT2R signaling can influence TAA development. Thus, AT1R blockade with losartan can protect against TAA in a *Fbn1*^C1039G/+^ mouse model of MFS, through inhibition of ERK1/2 signaling mediated by AT2R signaling [287]. Other studies have also reported that the AT1R antagonist, losartan, can attenuate ANGII-induced aortic dilation in murine models of MFS [288,289].

There is also evidence for interaction between ANGII and TGF-β signaling in some genetic aortopathies. Murine models of LDS, including *Tgfbr1^+/^^−^* or *Tgfbr2^+/^^−^* mice, have been reported to show an upregulation of TGF-β ligand expression, which appears to be dependent on ANGII/AT1R signaling [290]. Use of a neutralizing antibody to TGF-β in a murine MFS model results in worse aortic dilation and a greater incidence of rupture after ANGII treatment [243,288].

A deficiency of the smooth muscle-specific isoform of α-actin (SM α-actin), ACTA2, is also associated with TAA formation after ANGII. The mechanism appears to involve both the generation of ROS and the NF-κB-dependent induction of AT1R [291]. The ANGII signaling may also contribute to development of cerebral aneurysms in patients with *ACTA2* mutations [292]. Mice deficient in fibulin-4 develop TAA, which can be prevented with the AT1R blocker losartan. A deficiency of Fbln4 in VSMCs is associated with activation of ERK1/2 and Smad2/3, which are blocked with losartan [293].

Other signaling pathways, e.g., AKT, may protect from the development of TAA in response to ANGII. The stimulation of VSMC with ANGII results in increased MMP-9 synthesis, which may contribute to ECM remodeling. Although the TGF-β pathway shares the downstream elements of ERK1/2 with ANGII, the inhibition of TGF-β signaling in a murine model did not alter MMP-9 production, indicating that ANGII is the main stimulus for MMP9 upregulation in the VSMC [289]. The expression of AKT2, but not AKT1, is reduced in human TAA and AKT2-deficient mice develop TAA, with upregulated MMP9 and reduced TIMP1 expression, apparently mediated by FOXO1-dependent transcriptional regulation [294]. In addition, mice deficient in MMP2 develop TAA with ANGII infusion, due to impairment in TGF-β/SMAD2/3 signaling and impaired LTBT1 cleavage, thereby losing apparent protective effect of TGF-β signaling [295]. There is also evidence of a role for EC ANGII/AT1R in pathogenesis of TAA. Although hypertension consequent upon ANGII stimulation is independent of EC AT1R function, the inhibition of EC AT1R is protective against TAA [284].

Several lines of evidence suggest a role for ANGII/AT1R in pathogenesis of human TAA. Single nucleotide polymorphisms in *AT1R* gene have been associated with increased aortic stiffness [296]. In pathology studies, human TAA show increased miR-29 levels and miR-29 has been shown to promote ANGII mediated TAA formation in murine models [297]. Thus, the increase in miR-29 in human tissue may represent a compensatory response.

Following experimental findings that the AT1R blocker may attenuate TAA formation in MFS mice [239], multiple clinical trials have examined effect of treatment with AT1R blockers on the progression of TAA in MFS. An initial observational study suggested treatment with Losartan was beneficial [298]. Subsequent studies have yielded conflicting results, with two randomized studies showing no clear benefit of AT1R blockers compared to beta-blockers in MFS [299,300]. In contrast, two clinical trials have shown the benefit of adding AT1R blockers to beta-blockers in MFS, with a reduction in TAA growth rates [301,302,303]. These clinical findings are consistent with a role for ANGII/AT1R in the pathogenesis of TAA.

The evidence, experimental and clinical, supporting the role(s) of each of the signaling pathways in pathogenesis of TAA is summarized in Table 2.

## 10. Potential Therapeutic Interventions

Information on potential therapeutic interventions in TAA is limited, however evidence from other fields, particularly cancer treatment, points towards options for future investigation.

### 10.1. TGF-β/BMP/SMAD Pathway

An inhibitor of TGF-β and downstream signaling mechanisms, pirfenidone, was FDA approved in 2014 for the treatment of idiopathic pulmonary fibrosis, indicating the therapeutic potential of inhibiting TGF-β signaling in a clinical setting [304,305].

Several major classes of TGF-β inhibitors are currently being tested in clinical trials and include antisense oligonucleotides which cause degradation of TGF-β mRNA, TGF-β antibodies and ligand traps which prevent TGF-β binding to receptors, and small inhibitor molecules that inhibit TGF-β receptor kinases [306]. Additionally, well established treatments already used clinically show potential for TGF-β inhibition. For example, metformin, a widely used drug in the treatment of diabetes, has been shown to suppress TGF-β signaling, therefore showing it has therapeutic potential for diseases where TGF-β signaling is increased [307].

TGF-β plays a major role in cancer progression and metastasis [308]. Intriguingly, TGF-β exhibits similar traits in the cancer tumor microenvironment as in TAA, with the promotion of ECM remodeling, angiogenesis and fibrosis [308,309,310]. Therefore, numerous clinical trials to assess anti-TGF-β therapeutic’s in cancer treatment have been established and have been reviewed in detail previously [307]. Some examples of drugs which have entered clinical trials for the treatment of cancer include AP12009 (antisense oligonucleotide complementary to TGFβ2 mRNA), galunisertib, (a small-molecular inhibitor of TGF-β receptor type 1 kinase (ALK5)) and dalantercept (a ligand trap, binding to BMP9 and BMP10, therefore inhibiting ALK1 activation and downstream signaling). While some cancer trials are still ongoing and showing promising results [311,312,313,314], there have also been inconsistencies with the use of TGF-β inhibitors and clinical outcomes in cancer treatment [308,315]. These common inconsistency’s highlight the major challenge of inhibiting TGF-β due to its wide distribution in different tissue and its multiple key cellular functions [36].

Therapeutics targets of TGF-β in cancer are frequently being tested in combination with other cancer treatments to improve therapeutic potential. This need for TGF-β inhibitors in combination with other agents may also be relevant to the treatment of TAA, as the interactions between and redundancy of signaling pathways may mean the inhibition of TGF-β alone may not result in an effective outcome. This concept has been supported by the finding that TGF-β1 inhibitors combined with PI3K inhibitors are the most effective method for inhibiting the phenotype switch of human aortic VSMCs compared to the use of a single inhibitor [231].

### 10.2. WNT Pathway

Several drugs targeting the WNT pathway are in the clinical trial phase of testing, predominately for the treatment of cancer. These include inhibitors of WNT protein secretion, competitive antagonists of WNT binding to Frizzled, monoclonal antibodies to Frizzled and antagonists of Dishevelled binding to Frizzled [316].

Molecules targeting WNT secretion have shown promising results. For example, several inhibitors of porcupine, a protein that is essential for secretion of WNT, results in the inactivation of WNT ligands and the reduction of downstream signaling, arresting cell growth in preclinical models, with clinical trials now in progress for the treatment of solid tumors [317,318,319]. Another group of molecules emerging as potential cancer therapeutics are tankyrase inhibitors, which prevent the destruction of Axin, therefore reducing canonical WNT signaling. Preclinical studies in several cancer models show promising results, however, no tankyrase inhibitors are currently in clinical trials.

These molecules currently in development for cancer therapies also demonstrate clinical utility for the treatment of CVDs. The administration of a porcupine inhibitor after myocardial infarction in mice reduced adverse cardiac remodeling and fibrosis [320]. Additionally, the tankyrase inhibitor, XAV939, has shown to repress ROS generation in VSMCs as well as regulate VSMC proliferation, migration and apoptosis in a carotid artery ligation model [84].

While currently there are no FDA approved WNT pathway inhibitors, the repurposing of established agents has been an area of interest, with several FDA approved drugs shown to inhibit components of the WNT pathway [321]. Thus, aspirin is associated with an increased expression of the WNT antagonist DKK-1 [322]. Other nonsteroidal anti-inflammatory drugs including indomethacin, celecoxib and sulindac have also been shown to reduce WNT pathway activity [323,324]. Although inhibitors targeting WNT ligand and β-catenin have been shown to suppress overactivated WNT signaling in cancer, due to the ubiquitous nature of WNT signaling and complex crosstalk with other pathways, it is difficult to target specific cells [321,325].

### 10.3. PI3K/AKT Pathway

The PI3K/AKT pathway and the therapeutic potential of its inhibition has been extensively investigated in cancer. Numerous drugs inhibiting the PI3K/AKT pathway have been develop and progressed to human clinical trials. The inhibition of PI3K/AKT and/or mTOR strongly influence other pathways such as MAPK, downstream FOXO3a, WNT/beta-catenin and NOTCH [326], which may each play a role in TAA pathogenesis.

There are two main classes of PI3K/AKT inhibitors. The first are pan-PI3K inhibitors which do not discriminate between the different PI3K isoforms and were the first generation of inhibitors to be developed. One of these drugs includes wortmannin, which reduced aneurysmal growth when administered in an experimental animal model of AAA [327]. While this class of inhibitors have been widely used to uncover the importance of the PI3K pathway in several pathologies, there use has been associated with a range of side effects and toxicity due to their low selectivity [328]. Therefore, isoform-specific compounds which only target one or two isoforms exclusively were developed. There are currently three PI3K inhibitors that are FDA approved for the treatment of various cancers; idelalisib, copanlisib and duvelisib, which are all isoform specific compounds [326]. This suggests that selective PI3K inhibitors may be the most applicable in a clinical setting and this should be considered in the development of their use in the treatment of other diseases. Pre-clinical animal studies have supported this in the context of CVD, with PI3K selective inhibitors in models of atherosclerosis, myocardial infarction, thrombosis, hypertension and heart disease showing therapeutic potential [329]. Therefore, gaining insight into the specific PI3K isoforms which are involved in TAA pathogenesis may be the key to translating the use of PI3K inhibitors to the treatment of TAA.

### 10.4. NOTCH Pathway

Disordered NOTCH signaling is a feature of many cancers and a variety of targeted therapies have been developed, including inhibitors of the cleavage of NOTCH receptor precursors and gamma-secretase inhibitors [122]. The gamma-secretase inhibitors are the most well studied small-molecule inhibitors of NOTCH, which have shown antitumor effects in preclinical studies, with multiple agents progressing to clinical trials [330]. However, the lack of specificity of these inhibitors and associated toxicities has encouraged further development of more specific NOTCH inhibitors. More targeted interventions directed at specific receptor/ligand types may be effective and antibodies against JAG1 have been examined in cancer therapy [331]. Monotherapy has not proven very effective, due to crosstalk between RAS/MEK/ERK, PI3K/AKT and Hedgehog and therefore combination therapy may be required [331,332].

As cardiovascular abnormalities appear to be a feature of impaired NOTCH signaling, NOTCH agonists may be a useful therapeutic tool. However, given the fundamental role of NOTCH in vascular homeostasis, the overall inhibition of the pathway by nonspecific agents are unlikely to be beneficial. Therefore, the more targeted inhibitors currently being investigated in the treatment of cancer may be more useful in the treatment of CVD.

There have been some preclinical studies on abdominal aortic aneurysm using NOTCH inhibitors. In a murine model using ANGII infusion, aortic inflammation and elastin fragmentation were reduced by treatment with a NOTCH inhibitor [333,334]. Whether these findings may apply to TAA remains to be shown, particularly as the inflammatory response in TAA is considerably less than in abdominal aortic aneurysm.

### 10.5. Angiotensin II Pathway

The ANGII pathway is a well-studied treatment target in CVD, including in TAA. Angiotensin receptor blockers (ARBs) and beta-blockers have each been proposed to reduce TAA growth rates in MFS and other genetic aortopathies [289,335]. The AT1R blocker, losartan, has been investigated as a treatment for aortic dilatation in MFS and LDS, principally to suppress adverse consequences of AngII signaling. There may, however, be other mechanisms of benefit, independent of AngII blockade. A losartan metabolite EXP3179 has been shown to activate AKT and eNOS in EC and to suppress tumor necrosis factor α–induced EC apoptosis [336].

The magnitude of effect on slowing TAA growth is debated and available clinical studies do not all clearly differentiate individual effects of beta-blockers and ARB, as they may be used in combination. Data from experimental animal studies appear more robust than from clinical studies and the benefits of ARB use may be dependent upon the age of the treatment subject. In addition, other variables in human populations may impact the benefit of ARBs, including variability in underlying mutations causing MFS or LDS, epigenetic factors and drug dose and type [335]. Notwithstanding, a recent meta-analysis of available trials concludes that there is benefit of AT1R blockers in slowing the rate of aortic dilatation in MFS [303].

### 10.6. Application of Pathway Therapeutics to TAA

In summary, the work done on investigating therapeutic targets of TGF-β, WNT, PI3K/AKT, NOTCH and ANGII signaling in the treatment of cancer highlight the potential for these pathways to be targeted in the treatment of TAA. However, there are a few factors that need to be considered when beginning to translate the work done in cancer to their use in the context of TAA.

Firstly, several compounds which target these pathways show levels of toxicity when administrated for the treatment of cancer as the aggressive nature of the disease may require relatively high doses of a particular drug. However, this does not rule out their potential clinical utility in the treatment of TAA as it does not need to be approached in the same aggressive manner and therefore lower doses may be acceptable in TAA treatment, attenuating toxicity and potential side effects.

Secondly, the aggressive nature of cancer may, on the other hand, mean certain drugs are not potent enough to show any advantageous therapeutic effect. However, a small effect on any of these pathways may be enough to influence TAA development and therefore may be beneficial in TAA treatment and are still worth pursuing.

Finally, therapeutic targets of the five pathways discussed in cancer are frequently being tested in combination with other cancer treatments to improve therapeutic potential. This need for TGF-β inhibitors in combination with other agents may also be relevant to the treatment of TAA, as the interactions between and redundancy of signaling pathways may mean inhibition of TGF-β alone may not result in an effective outcome. This concept has been supported by the finding that TGF-β1 inhibitors combined with PI3K inhibitors are the most effective method for inhibiting the phenotype switch of human aortic VSMCs compared to the use of a single inhibitor [231].

## 11. Conclusions

Multiple signaling pathways influence both EC and VSMC phenotype, often acting in synergy to either promote or inhibit cell proliferation and phenotype. Crosstalk between two or more pathways in determining cell fate appears to be common. Signaling outcomes consequent upon the activation of a specific pathway EC and VSMC may, however, differ and each cell type has the capacity to influence outcomes in the other.

At this time a cognate integrative description of the mechanism of TAA formation remains elusive. There is evidence indicating potential for each of the major pathways described to contribute to TAA formation. Experimental and human studies support the roles of these pathways in TAA, however the evidence base remains fragmentary and further work is required, particularly in the field of signaling cross-talk.

For many patients with TAA, the underlying cause remains unknown, including for many with non-syndromic h-TAA. It is quite likely that many of these patients have an underlying dysfunction/dysregulation of the signaling pathways described and an understanding of the key regulatory points in these pathways may illuminate potential new loci for the investigation of gene mutations in TAA.

Despite great interest, there is very limited data on efficacy of novel treatments for prevention of TAA. The experience in cancer medicine illustrates potential new approaches, but at the same time also highlights the challenges of drug toxicity and off-target effects. The multiplicity of pathways influencing cell fate infer redundancy and, therefore, therapeutic attempts to influence a single pathway may be sub-optimal in altering TAA development. Although much work remains to be done, our improved understanding of the roles of multiple cell-signaling pathways upon cell fate in the vasculature can provide a foundation for future advances.

## Figures and Tables

**Table 1:** Summary table of evidence for direct and/or indirect effects of pathways in promoting different cellular responses in VSMC and EC. + equals effect observed.

**Vascular Smooth Muscle Cell**						
Pathway	Promote contractile phenotype and survival		Promote proliferative response		Promote synthetic phenotype, apoptosis, proliferation, and migration	
	Direct	Indirect	Direct	Indirect	Direct	Indirect
TGF-β	+		+	+	+	
WNT	+		+		+	
PI3K/AK	+	+			+	
NOTCH	+	+	+		+	
ANGII					+	+
Endothelial Cell						
Pathway	Promote survival and EC quiescence		Promote proliferation and migration		Promote EC dysregulation, apoptosis, and inflammation	
	Direct	Indirect	Direct	Indirect	Direct	Indirect
TGF-β	+		+	+		
WNT	+	+	+	+	+	
PI3K/AK	+	+	+	+		
NOTCH	+					
ANGII		+			+	+

**Table 2:** Summary of evidence for signaling pathways in pathogenesis of thoracic aortic aneurysm.

Pathway	Experimental	Human
TGF-ß/SMAD	Murine Murine MFS/LDS	Isolated aortic VSMC Proteomics Clinical Genetics
Angiotensin II	Murine Murine MFS	Clinical
PI3K/AKT	Murine Murine MFS	Isolated aortic VSMC TAA tissue
WNT	Murine Murine MFS Aortic interstitial cells	Cardiac fibroblasts Isolated EC Isolated aortic VSMC GWAS TAA tissue
NOTCH	Murine Murine MFS	Isolated EC Isolated aortic VSMC Proteomics TAA tissue Clinical Genetics

## Data Availability

Not applicable.

## Abbreviations

ACVRL1	activin A receptor like type 1
ADAM17	A disintegrin and metalloproteinase 17
ADNP	activity dependent neuroprotector homeobox transcription factor
ALK	ALK receptor tyrosine kinase
AKT	protein kinase B
ANG	Angiotensin
AP-1	activator protein 1
AT1(2)R	angiotensin type 1 (2) receptor
BAD	BCL2 associated agonist of cell death
BAEC	bovine aortic endothelial cell
BAMBI	BMP and activin membrane bound inhibitor
BAV	bicuspid aortic valve
BMP	bone morphogenic protein
CADASIL	cerebral autosomal dominant arteriopathy with sub-cortical infarcts and leukoencephalopathy
CAMKII	calcium/calmodulin dependent protein kinase 2
CCND1	cyclinD1
COX2	cyclooxygenase 2
CREB	cAMP response element binding protein transcription factor
CSL/RBPJ	recombination binding protein for immunoglobulin Kappa J
CTGF	connective tissue growth factor
DKK	Dickkopf WNT signaling pathway inhibitor
DLL	delta like canonical Notch ligand
DVL1	disheveled segment polarity protein 1
EC	endothelial cell
ECM	extracellular matrix
EGFL7	epidermal growth factor like domain multiple 7
EGFR	epidermal growth factor receptor
ERK	extracellular signal-regulated kinase
FN1	fibronectin 1
FOXO	forkhead box gene group O
FZD	frizzled receptor
GSK-3β	glycogen synthase kinase 3 beta
H_2_O_2_	hydrogen peroxide
HERP	homocysteine inducible ER protein
HES	hairy and enhancer of split-1 transcription factor
HEY	hairy ears Y linked transcription factor
HAEC	human aortic endothelial cell
HUVEC	human umbilical vein endothelial cell
hTAA	heritable thoracic aortic aneurysm
IGFBP	insulin-like growth factor binding protein
IGF-1R	insulin-like growth factor 1 receptor
IL-8	interleukin 8
JAK2	janus kinase 2
JNK	c-Jun N-terminal kinase
LDL	low density lipoprotein
LDS	Loeys-Dietz syndrome
LEF	lymphoid enhancer binding factor transcriptional activator
LRP	low-density lipoprotein receptor-related protein
LTBP	latent TGFbeta binding protein
MAGP-2	microfibril associated glycoprotein 2
MAML	mastermind-like protein 1
MAPK	mitogen activated protein kinase
MAS	Mas-related G protein couple receptor
MDM2	MDM2 proto-oncogene E3 ubiquitin protein ligase
MEF2	myocyte enhancer factor 2C
MEK	MAPK/ERK kinase
MFS	Marfan syndrome
MK2	mitogen-activated protein kinase-activated protein kinase 2
MMP	matrix metalloproteinase
mTOR	mammalian target of rapamycin
NADPH	nicotinamide adenine dinucleotide phosphate
NEDD4	neural precursor cell expressed developmentally downregulated protein 4
NF-κB	nuclear factor kappa B
NICD	Notch intracellular domain
NO	nitric oxide
NOS	nitric oxide synthase
NOTCH	Notch receptor protein
NOTUM	Notum palmitoleoyl-protein-carboxylesterase
NOX	NADPH oxidase
PAI-1	plasminogen activator inhibitor type-1
PDGF	platelet-derived growth factor
PI3K	phosphoinositide 3-kinase
PIP_3_	phosphatidyinositol (3,4,5)-triphosphate
PKC-δ	protein kinase C gamma
PLA(C)	phospholipase A (C)
PTEN	phosphatase and tensin homolog
p38	p38 mitogen-activated protein kinase
ROCK	Rho associated coiled-coil containing protein kinase
ROS	reactive oxygen species
RTK	receptor tyrosine kinase
RUNX	RUNX family transcription factor
SARA	SMAD anchor for receptor activation
SKI	SKI proto-oncogene
SM22α	smooth muscle protein 22-alpha
SMA	smooth muscle actin
SMAD	small mothers against decapentaplegic
SMURF	SMAD ubiquitination regulatory factor
SNON	SKI-like proto-oncogene
SOD	superoxide dismutase
SOST	Sclerostin
SP1	specificity protein 1 transcription factor
SPRY1	sprouty RTK signaling antagonist 1
TAA	thoracic aortic aneurysm
TAV	tricuspid aortic valve
TCF	T-cell factor enhancer binding factor transcriptional activator
TGF-β	transforming growth factor β
TGFBR	transforming growth factor beta receptor
TIMP1	tissue inhibitor of metalloproteinases 1
TNF-α	tumor necrosis factor alpha
TRABD2B	TraB domain containing 2B
VCAM-1	vascular cell adhesion molecule 1
VCAN	Versican
VEGF	vascular endothelial growth factor
VSMC	vascular smooth muscle cells
WIF-1	WNT inhibitory factor 1
WISP	WNT1 inducible signaling pathway protein
WNT	wingless-related integration site
ZNRF	zinc and ring finger ubiquitin protein ligands
